# Cardiac comorbidities in McArdle disease: case report and systematic review

**DOI:** 10.1007/s10072-024-07600-x

**Published:** 2024-05-27

**Authors:** Domeniko Hoxhaj, Gabriele Vadi, Lorenzo Bianchi, Lorenzo Fontanelli, Francesca Torri, Gabriele Siciliano, Giulia Ricci

**Affiliations:** 1 Department of Clinical and Experimental Medicine, Neurological Institute, University of Pisa, Via Roma 67, 56100 Pisa, Italy; 2 Department of Internal Medicine, University of Genova, Genoa, Italy

**Keywords:** McArdle disease, Myophosphorylase deficiency, Cardiac comorbidities, Coronary artery disease

## Abstract

**Introduction and methods:**

Myophosphorylase deficiency, also known as McArdle disease or Glycogen Storage Disease type V (GSD-V), is an autosomal recessive metabolic myopathy that results in impaired glycogen breakdown in skeletal muscle. Despite being labelled as a “pure myopathy,” cardiac involvement has been reported in some cases, including various cardiac abnormalities such as electrocardiographic changes, coronary artery disease, and cardiomyopathy. Here, we present a unique case of a 72-year-old man with GSD-V and both mitral valvulopathy and coronary artery disease, prompting a systematic review to explore the existing literature on cardiac comorbidities in McArdle disease.

**Results:**

Our systematic literature revision identified 7 case reports and 1 retrospective cohort study. The case reports described 7 GSD-V patients, averaging 54.3 years in age, mostly male (85.7%). Coronary artery disease was noted in 57.1% of cases, hypertrophic cardiomyopathy in 28.5%, severe aortic stenosis in 14.3%, and genetic dilated cardiomyopathy in one. In the retrospective cohort study, five out of 14 subjects (36%) had coronary artery disease.

**Discussion and conclusion:**

Despite McArdle disease primarily affecting skeletal muscle, cardiac involvement has been observed, especially coronary artery disease, the frequency of which was moreover found to be higher in McArdle patients than in the background population in a previous study from a European registry. Exaggerated cardiovascular responses during exercise and impaired glycolytic metabolism have been speculated as potential contributors. A comprehensive cardiological screening might be recommended for McArdle disease patients to detect and manage cardiac comorbidities. A multidisciplinary approach is crucial to effectively manage both neurological and cardiac aspects of the disease and improve patient outcomes. Further research is required to establish clearer pathophysiological links between McArdle disease and cardiac manifestations in order to clarify the existing findings.

**Supplementary Information:**

The online version contains supplementary material available at 10.1007/s10072-024-07600-x.

## Introduction

Myophosphorylase deficiency, also known as McArdle disease or Glycogen Storage Disease type V(GSD-V), is the most common metabolic myopathy and was firstly described in 1951 by Dr. Brian McArdle, who reported the case of a man with a history of exercise induced cramps [1]. This autosomal recessive disorder arises due to mutations in *PYGM* gene on chromosome 11q13, causing a deficiency of the myophosphorylase enzyme, which plays a crucial role in the breakdown of glycogen in skeletal muscle cells [2]. This rare condition leads to the impaired ability of affected individuals to access stored glycogen as an energy source during physical activity, resulting in a distinct set of clinical features. Although some individuals may remain asymptomatic until adulthood, symptoms onset generally occurs during adolescence, characterized by exercise intolerance which presents with fatigue, pain, and contractures after few minutes of exercise. Interestingly, individuals with McArdle disease may experience a remarkable characteristic known as the “second-wind” phenomenon, which refers to an improvement in exercise tolerance and a reduction in muscle symptoms after a brief period of rest during physical activity. CK levels are usually elevated in these patients and episodes of rhabdomyolysis may as well occur at some point in their lives [3].

Unlike many other myopathies, McArdle’s disease is not typically associated with multisystem involvement and some reviews have labelled this condition as a “pure myopathy”, since the muscle isozyme myophosphorylase is also present in other tissue such as the heart and the brain. However, in these tissues, the myophosphorylase deficiency is adequately compensated for by the substantially higher expression of the brain isozyme [4]. Nevertheless, a few cases of cardiological involvement have been described. In particular, reported cardiac manifestations of McArdle disease include electrocardiographic changes, coronary artery disease, and hypertrophic cardiomyopathy [5]. We report the case of a 72-year-old man with McArdle disease, early mitral and tricuspid valve disease and coronary artery disease, that is a rare combination of cardiac comorbidities, who underwent both tricuspid and mitral annuloplasty surgeries, as well as coronary angioplasty with the placement of four stents. Considering the limited number of reported cases of cardiological abnormalities in McArdle disease, we have opted to conduct a systematic review to analyse the existing findings in order to explore a possible link between the two conditions.

## Illustrative case

**Fig. 1:**
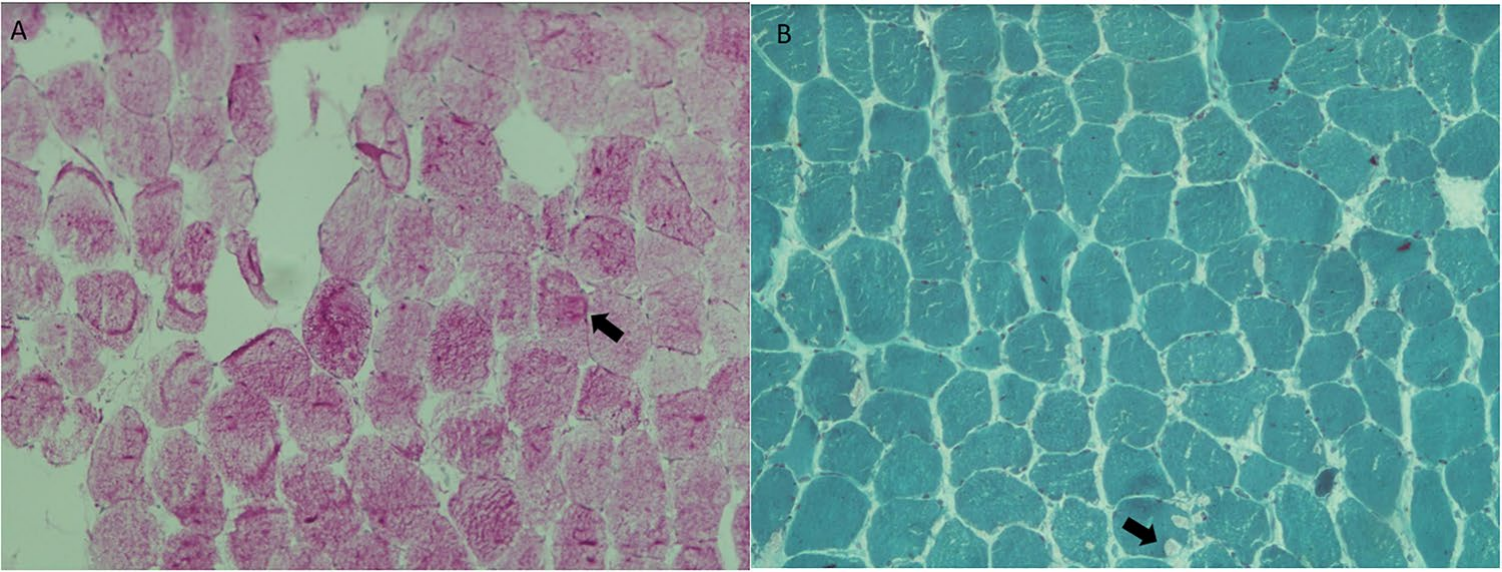
Muscle biopsy from our case (left quadriceps femori). **A** 10x, PAS staining; **B** 10x, Gomori's trichrome staining. Arrows indicating glycogen vacuoles

**Fig. 2:**
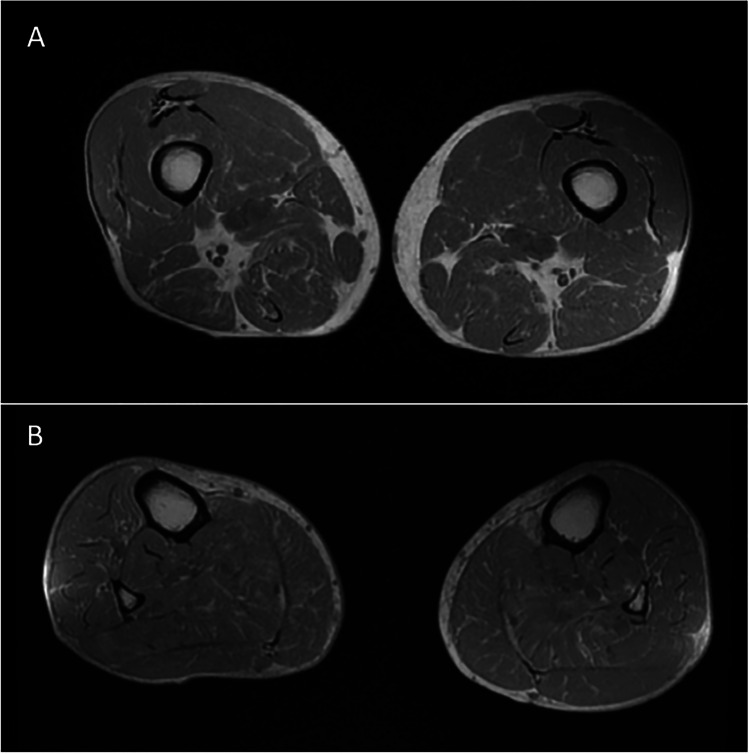
Ax IN/OUT MRI sequences of our case showing signs of fatty infiltration in the posterior compartment of the thighs (**A**) and in the anterior and posterior compartment of lower legs (**B**)

A 72-year-old man with a history of mild hypertension and dyslipidaemia is regularly followed at Neuromuscular Unit of Santa Chiara Hospital in Pisa for the diagnosis of McArdle disease. He does not smoke and engages in regular physical activity. The patient's first evaluation at our clinic occurred when he reached 64 years of age, for a long-standing history of exercise intolerance that has been present since his adolescence. He never complained muscle weakness, nor he reported episodes of myoglobinuria or rhabdomyolysis. The "second-wind" phenomenon was clearly described by the patient since early adulthood. At that time we performed an ischemic forearm test that showed a blunted lactate response post-exercise and a muscle biopsy from quadriceps femoris that revealed a vacuolar myopathy with an increase of glycogen and absent myophosphorylase activity (Fig. 1). Genetic analysis confirmed the diagnosis of McArdle disease showing the common homozygous p.Arg50 stop codon mutation in *PYGM* gene. Over time, his muscle symptoms remained stable, and his average CK levels stayed within the range of 200–350 U/L, with a peak reaching 3500 U/L. A lower limbs muscular magnetic resonance imaging (MRI) showed signs of fatty infiltration in the anterior and posterior compartment of lower legs, in the posterior compartment of the thighs, gluteus, and paravertebral muscles (Fig. 2). During the last follow-up the neurological examination showed a hyperlordotic gait, a mild bilaterally deltoids hyposthenia (MRC 4), pectoral muscles hypotrophy, and preserved strength in all other muscle groups. His cardiological history begins at the age of 35, when a mitral valve prolapse with mild regurgitation was detected. Electrocardiogram was normal and blood pressure values showed only a mild hypertension. Since then, regular instrumental and clinical follow-up showed no significant changes until the age of 61, when he presented dyspnoea (New York Heart Association class 3) due to progression of mitral regurgitation from mild to severe, along with the concomitant finding of a mild tricuspid regurgitation. As a result, he underwent mitral and tricuspid annuloplasty surgery. During the procedure, a coronary angiography revealed significant stenosis in the marginal branch of the circumflex artery and multiple subcritical stenoses in the right coronary artery. Due to the absence of angina, coronary revascularization was not performed at that time. Subsequent cardiological follow-ups, including cycle ergometer exercise tests and myocardial scintigraphy, were periodically conducted without evidence of inducible myocardial ischemia. At 72 years of age, the patient was admitted for elective coronary angiography due to exertional dyspnea, which was interpreted as angina equivalent given the context of pre-existing two-vessel coronary artery disease. Echocardiography showed good overall systolic function with basal inferior wall akinesia and medio-basal posterolateral hypokinesia, a good functioning of the mitral and tricuspid valves, and mild aortic insufficiency. The coronary angiography revealed widespread atherosclerosis and identified two significant stenoses (80%) in the proximal and middle segments of the circumflex artery, as well as critical stenoses in the middle of the right coronary artery (80%) and its posterolateral branch (90%). Angioplasty was then performed, successfully placing two drug-eluting stents in the circumflex artery and an additional two in the right artery. No issues were reported during the percutaneous coronary intervention, performed under local anaesthesia with mepivacaine. As part of secondary prevention and given the presence of myopathy, the patient was prescribed alirocumab, a PCSK9 inhibitor. This therapy efficiently regulated his LDL cholesterol levels and was overall well-tolerated. Subsequent cardiological follow-ups have shown no concerning developments.

## Methods

The study was conducted following the Preferred Reporting Items for Systematic Reviews (PRISMA) guidelines [6]. A systematic literature search was conducted using PubMed/MEDLINE until July 2023. No language or country of publication filters were applied to retrieve all available literature. The complete search string was: ("McArdle Disease " OR "Glycogen Storage Disease Type V" OR "myophosphorylase deficiency" OR "GSD type V" OR "McArdle ") AND (''card'' or "cardiovascular diseases" or ''cardiac involvement '' or ''angina'' or ''valvulopathy'' or ''valvular heart disease'' or ''cardiomyopathy'' or ''coronaropathy'' or ''coronary artery disease'' or ''CAD”). Screening and eligibility assessment of articles was performed in Rayyan – a web and mobile app for systematic reviews (https://www.rayyan.ai).

Inclusion criteria were: studies (1) containing at least one well described case of McArdle disease with cardiac comorbidities, (2) discussing McArdle syndrome and its comorbidities, encompassing cardiac involvement or potential pathophysiological rationales for such associations.

Exclusion criteria were: (1) review on McArdle in general, (2) studies without detailed information about individual patients.

The articles were searched and extracted by two reviewers (D.H. and G.V.), and any discrepancies were solved by a third investigator (L.B.). We pulled out several variables including age, sex, clinical onset presentation, clinical neurological features, lactate test, CK level, muscle MRI, muscle biopsy, genetic mutation, cardiac comorbidity, electrocardiogram (ECG), echocardiogram (ECHO), cardiac MRI, coronary angiography, presence of other comorbidities. Continuous variables were presented as means ± standard deviations while categorical variables were presented as absolute values and percentages. Microsoft Excel was used to extract data and perform calculations. The collected results from the case reports were summarized in tables (Tables 1 and 2). The quality of the studies included was assessed using the Joanna Briggs Institute Critical Appraisal [7]. One reviewer (B.L) assessed each article and then assigned a consensus score to each. Score reports are provided in the supporting information: Tables S2–S3. Due to the heterogeneity in result description, study design, and participant selection, the available data were qualitatively described, and no meta-analysis was performed.

**Table 1:** Demographics and neuromuscular features of the cases

Sr. No	Article type	References	Country	Number of patients	Age (years) Gender (M/F)	Clinical neurological features	Lactate test	CK levels	Muscle biopsy	Genetic analysis (*PYGM* gene)
1	Case report	Nicholls et al. 1996 [8]	Ireland	1	66 M	Muscle pains on exertion since teenager	Not mentioned	> 1000 U/L	Absent myophosphorylase activity, increase in glycogen	Nonsense mutation in exon 1
2	Case report	Moustafa et al. 2012 [9]	Canada	1	33 M	Muscle fatigue, cramps, exercise intolerance	Flat lactate curve, increased ammonia	Persistently > 5000 U/L	Not mentioned	p.Arg50* mutation
3	Case report	Marco-Benedí et Al. 2019 [10]	Spain	1	60 M	Myalgia and exercise intolerance since childhood	Not mentioned	> 10,000 U/L	Not mentioned	Not mentioned
4	Case report	Jones et al. 2019 [5]	UK	1	69 M	Not mentioned	Not mentioned	Not mentioned	Not mentioned	Homozygous p.Arg50* mutation
5	Case report	Vavouranakis et al. 2007 [11]	Greece	1	62 M	Slight fatigue for long muscular efforts	Flat lactate curve, normal ammonia	2680–4400 U/L	Absent myophosphorylase activity, increase in glycogen	Not mentioned
6	Case report	Lepoivre et al. 2022 [12]	France	1	29 F	Leg muscles pain while walking	Not mentioned	809 U/L	Absent myophosphorylase activity	Not mentioned
7	Case report	Wang et al. 2015 [13]	Australia	1	61 M	Not mentioned	Not mentioned	Not mentioned	Not mentioned	Not mentioned
8	Retrospective cohort study	Gandhi et al. 2021 [14]	Scotland	14	43.8 ± 16.1 8:6 M/F	Exercise-induced cramps, exercise intolerance (100%)	Performed in 7/13 (54%), flat lactate response in 7/7 (100%)	772 U/L as basal mean, 5000–128000 U/L as range during rha = bdomyolysis	Performed in 10/14 (71%), absent myophosphorylase activity in 10/10	93% had at least one copy of the p.Arg50* variant, 50% being homozygous

**Table 2:** Clinical presentation and cardiological main features of the cases

Sr. No	Article type	References	Clinical presentation of the case report	Cardiac comorbidity	ECG and Echocardiographic data	Coronary angiography and treatment	Other comorbidities
1	Case report	Nicholls et al. 1996 [8]	Effort angina	CAD	Not mentioned	Severe triple vessel occlusion treated with CABG	Hypertension, Gout
2	Case report	Moustafa et al. 2012 [9]	Incidental systolic murmur detection	HCOM	Asymmetrical septal hypertrophy, systolic anterior motion of the mitral valve with severe LVOTO, significant mitral regurgitation, normal LV systolic function	Not mentioned	Obesity
3	Case report	Marco-Benedí et al. 2019 [10]	Rhabdomyolysis associated with statins as treatment after myocardial infarction	Myocardial infarction	Not mentioned	Not mentioned	DM2, familial combined hyperlipidemia
4	Case report	Jones et al. 2019 [5]	Atypical non-exertional chest pain with a satisfactory exercise tolerance	HCM, CAD	Inferolateral T-wave flattening, precordial late progression QRS; normal EF, asymmetric septal hypertrophy, areas of hypoperfusion and fibrosis	Multivessel CAD, moderate residual distal LAD artery disease after treatment with PCI	Not mentioned
5	Case report	Vavouranakis et al. 2007 [11]	Persistent hyperCKemia associated with statin as treatment after myocardial infarction	ACS treated with percutaneous revascularization	Not mentioned	ACS due to stenosis of the circumflex artery treated with PCI	None
6	Case report	Lepoivre et al. 2022 [12]	Dyspnea during second pregnancy	Genetic CMD	Worsening of ventricular function during pregnancy. Mild to moderate left ventricular dysfunction, mild-moderate mitral regurgitation	Not mentioned	Not mentioned
7	Case report	Wang et al. 2015 [13]	Symptoms due to severe aortic stenosis	Severe aortic stenosis treated with TAVI, then PM and SAVR	Severe aortic stenosis, bicuspid valve, mild left ventricle hypertrophy	Not mentioned	Hypertension, dyslipidemia, obesity (BMI 42), OSAS
8	Retrospective cohort study	Gandhi et al. 2021 [14]	Not mentioned	CAD 5/14 (35.7%)	Not mentioned	Not mentioned	7% (1/14) renal failure due to myocardial infarction; 29% (4/14) hypertension

*Abbreviations*: *ACS* Acute coronary syndrome, *CABG* Coronary-artery bypass graft, *CAD* Coronary artery disease, *CMD* Dilated cardiomyopathy, *DM* Diabetes mellitus, *EF* Ejection fraction, *HOCM* Hypertrophic obstructive cardiomyopathy, *LAD* Left anterior descending, *LVOTO* Left ventricle outflow tract obstruction, *PM* Pace-maker, *SAVR* Surgical aortic valve replacement, *TAVI* Trans-catheter aortic valve replacement

## Results

### Case reports

**Fig. 3:**
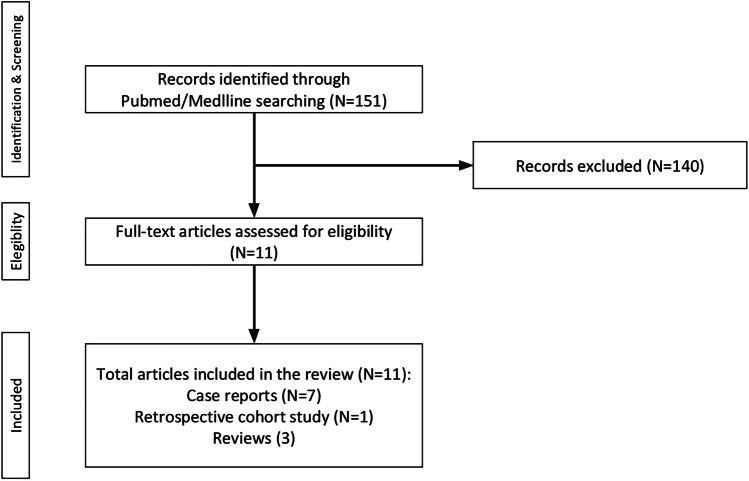
Flowchart showing the results of our literature search

The search identified 151 articles: among these, 140 were excluded due to their irrelevance to the topic, and from the remaining 11, after screening, 8 articles comprising 7 case reports and 1 retrospective cohort study were selected. The remaining articles were included in the discussion part (Fig. 3).

Data from seven patients were described in the form of case reports (Tables 1, 2). The average age of the patients was 54,3 ± 16,2 years (range: 29–69 years). Six patients (85.7%) were male, while only one patient was a female (14.3%). Four out of seven patients (57.1%) presented with significant acute or chronic coronary artery disease requiring percutaneous intervention (PCI) [5, 11] or surgical revascularization by aorto-coronary bypass (CABG) [8], while in one case no specific treatment was mentioned [10]. Two patients had hypertrophic cardiomyopathy (28.5%), one being a severe obstructive form [9], while the other consisting of a non-obstructive form with concomitant significant coronary artery disease (CAD) and ECG abnormalities [5]. One patient had severe aortic stenosis in a bicuspid valve (14.3%) which required transcatheter valve replacement; the intervention was complicated two days later by a type 2 atrioventricular block necessitating a pacemaker insertion [13]. Lastly, one patient had a genetic (not otherwise specified) dilated cardiomyopathy, with clinical and functional deterioration during pregnancy, resulting in premature cesarean section performed under general anesthesia [12].

Among the examined case reports, the onset of cardiological symptoms in relation to the diagnosis of myopathy was not specified for one patient, while in three out of the six remaining patients, cardiological symptoms presented before the diagnosis of myopathy. In one patient the diagnosis of myopathy was made a few years after the diagnosis of dilated cardiomyopathy, both diseases occurring in young adulthood [12]. Interestingly, in two out of six patients, the diagnosis of GSD-V was made due to the appearance of muscular symptoms and/or an increase in CK levels triggered by the initiation of statin therapy following acute coronary syndrome [10, 11].

Regarding the most frequent musculoskeletal symptoms, muscle fatigue and exercise intolerance, with or without cramps, were reported in all patients, while rhabdomyolysis with myoglobinuria was reported in one case only [10]. CPK levels, when available (six out of seven patients), were higher than the normal range in all patients (Tables 1, 2).

### Observational study

The retrospective Scottish cohort study included a total of 14 patients (8 males and 6 female) with GSD-V. Participants had a mean age at clinical presentation for myopathy of 43.8 ± 16.6 years with genetic diagnosis established at an average age of 47.7 ± 16.1. Five out of 14 subjects (36%) had coronary artery disease. However, clinical presentations, onset age, and information concerning any revascularization procedures were not specified. All 14 patients had exercise intolerance and muscle cramps, 12 patients (86%) suffered from myalgia, and 10 (71%) manifested the "second-wind" phenomenon. Episodes of rhabdomyolysis and myoglobinuria occurred in 10 patients (71%), and elevated CPK levels were observed in all patients [14].

## Discussion

McArdle disease is a metabolic myopathy that does not specifically affect cardiac muscle [4]. However, even though a sufficient myocardial myophosphorylase activity is expected to counterbalance the absence of the skeletal muscle isoenzyme, some cases of cardiac involvement have been reported as a manifestation of the disease itself [5]. To our knowledge, this is the first case of a double annuloplasty surgery with simultaneous finding of coronary artery disease in a patient with McArdle disease, which, a decade later, required an angioplasty procedure with the insertion of four drug eluting stents across distinct arteries. Both surgical procedures were well-tolerated by the patient and no specific anhestesia-related complications were reported. Only one case of hyperthermia, pulmonary oedema and rhabdomyolysis after protamine administration was reported in literature in occurrence with McArdle disease [15], however a later review concluded that such myopathy doesn’t seem to be related to severe perioperative problems in routine anaesthetic care [16]. Of note, after the angioplasty procedure, considering the presence of muscular disease, the patient was prescribed a PCSK9 inhibitor with an effective control over LDL levels and an excellent tolerability profile, confirming what has been previously reported concerning the safety of such drugs in McArdle patients [10].

In our revision of the literature, coronary heart disease (CHD) represented the most frequent findings among cardiac comorbidities (57.1%), while lower prevalence was detected for hypertrophic cardiomyopathy (28.5%) and valvular heart disease (14.3%). Indeed, a European registry study [17] reported a frequency of CHD of 8,3% in a large cohort of patients with McArdle disease, pointing out that such prevalence is higher than the background population, where it is estimated to be 2,8% in Europe [18]. However, so far, no study has compared the prevalence or incidence of such comorbidity across different age groups. The authors of the European registry study emphasized the importance of preventive screening for coronary artery disease, in light of their results. Our research supports this significant study, as CHD is the most prevalent cardiac abnormality observed in the available case reports. Interestingly, it has been hypothesised that some of the cardiological findings in McArdle disease may be related to the exaggerated autonomic cardiovascular responses occurring during submaximal exercise due to low concentrations of Na + /K + pumps in muscle and the resulting increase in extracellular K + concentration [19]. Earlier, an article reported the case of electrocardiographic alterations, including first-degree atrioventricular block and T-wave abnormalities, in a young man with McArdle disease, speculating a possible role of the impaired glycolytic metabolism in the conducting system [20]. However, no evidence of such an infiltration in the heart has been reported so far in literature. No specific electrocardiographic findings were mentioned among the examined case reports expect for one case that reported left axis deviation, inferolateral T-wave flattening, and late progression of QRS in the precordial leads [5], and another case with a type 2 atrioventricular block likely due to a post-operative complication [13].

Our patient was discovered with coronary artery disease at the age of 61, however it’s worth noting that his first signs of cardiac abnormalities dates back at the age of 35. The mean age of the available case reports is 54,3 ± 16,2 years and 43,8 ± 16,1 years for the retrospective study. While our case reports include younger patients with McArdle disease, we cannot conclude that the age of onset of cardiac abnormalities, although reported [17] more frequently in the general population, necessarily occurs at an earlier age compared to the general population. Of note, our patient reported regular physical activity but we can’t exclude that sedentary lifestyle related to pain experienced during exertion and exercise intolerance associated with McArdle disease might play a role in the development of coronary heart disease.

Regarding the possible role of biomarkers, in a review conducted by Molares-Vila et al. [21] no specific serum biomarker for McArdle disease, nor for a possible cardiological involvement were found.

While the pathophysiology of such involvement remains unproved, our work highlights the importance of a comprehensive cardiological screening in patients with McArdle disease given the variety of possible cardiological manifestations. Indeed, the proposed potential pathophysiological mechanisms linking the two conditions, and most importantly, the reported increased prevalence of coronary artery disease in McArdle patients, suggest a possible link between McArdle disease and cardiac involvement. Therefore, given our findings, we believe that considering a periodical check-up comprising electrocardiogram, transthoracic echocardiography, and cardiological examination would be beneficial in patients with McArdle disease, corroborating what previous research [17] highlighted concerning the importance of preventing comorbidities such as coronary artery disease in this condition.

Our study has several limitations. In particular, given the rarity of the disease, the available quantity of case reports and studies delving into the cardiac implications of McArdle disease remains limited. More extensive investigations involving larger cohorts of patients with a comprehensive cardiac evaluation should be conducted to gain deeper insights into the diverse phenotypic attributes of potential heart involvement. Additionally, the mechanisms underlying the connection between this cardiac involvement and glycogenosis remain largely unexplored. Preclinical research focusing on these aspects is therefore warranted and insights may be gleaned from larger and more detailed cohort studies.

## Conclusion

This systematic review sheds light on the relatively rare but significant association between McArdle disease and cardiac comorbidities. The findings suggest that individuals with GSD-V may be at increased risk of developing coronary artery disease and other cardiac conditions, warranting close cardiac monitoring. A multidisciplinary approach that integrates neurologic and cardiac assessments is crucial for the comprehensive management of patients with McArdle disease, allowing for early detection and personalized interventions to improve their overall health and well-being.

## Supplementary Information

**Additional file 1: Table_S2.** Quality Appraisal of Case Reports. **Table_S3.** Quality Appraisal of Original Articles.

## Data Availability

The data that support the findings of this study are available from the corresponding author upon reasonable request.

## References

[1] McARDLE B (1951) Myopathy due to a defect in muscle glycogen breakdown. Clin Sci 10(1):13–35 24540673

[2] Lucia A, Nogales-Gadea G, Pérez M, Martín MA, Andreu AL, Arenas J (2008) McArdle disease: What do neurologists need to know? Nat Clin Pract Neurol 4(10):568–577. 10.1038/ncpneuro0913 18833216 10.1038/ncpneuro0913

[3] Nogales-Gadea G, Santalla A, Brull A, de Luna N, Lucia A, Pinós T (2015) The pathogenomics of McArdle disease—genes, enzymes, models, and therapeutic implications. J Inherit Metab Dis 38(2):221–230. 10.1007/s10545-014-9743-2 25053163 10.1007/s10545-014-9743-2

[4] Di Mauro S (2007) Muscle glycogenoses: an overview. Acta Myol 26(1):35–41. https://www.ncbi.nlm.nih.gov/pmc/articles/PMC294932 PMC2949320 17915567

[5] Jones DM, Lopes L, Quinlivan R, Elliott PM, Khanji MY (2019) Cardiac manifestations of McArdle disease. Eur Heart J 40(4):397. 10.1093/eurheartj/ehy783. Oxford University Press 30534954 10.1093/eurheartj/ehy783

[6] Page MJ et al. (2021) The PRISMA 2020 statement: an updated guideline for reporting systematic reviews. BMJ 372. 10.1136/BMJ.N71 10.1136/bmj.n71 PMC8005924 33782057

[7] Munn Z et al (2020) Methodological quality of case series studies: an introduction to the JBI critical appraisal tool. JBI Evid Synth 18:10. 10.11124/JBISRIR-D-19-00099 10.11124/JBISRIR-D-19-00099 33038125

[8] Nicholls DP, Campbell NPS, Stevenson HP, Patterson VH (1996) Angina in McArdle’s disease. Heart 76(4):372–373. 10.1136/HRT.76.4.372 8983689 10.1136/hrt.76.4.372 PMC484554

[9] Moustafa S, Patton DJ, Connelly MS (2013) Unforeseen cardiac involvement in McArdle’s disease. Heart Lung Circ 22(9):769–771. 10.1016/J.HLC.2012.12.004 23337261 10.1016/j.hlc.2012.12.004

[10] Marco-Benedí V, Jarauta E, Pérez-Calahorra S, Bea AM, Civeira F (2019) Treatment of a high cardiovascular risk patient with McArdle’s disease with PCSK9 inhibitors. Clin Investig Arterioscler 31(2):89–92. 10.1016/J.ARTERI.2018.11.005 30738610 10.1016/j.arteri.2018.11.005

[11] Vavouranakis I, Ganotakis ES, Manta P, Evangeliou A (2007) Elevated creatine kinase levels in a patient with coronary artery disease and asymptomatic McArdle’s disease. Int J Cardiol 115(1):114–115. 10.1016/j.ijcard.2005.12.027 16762431 10.1016/j.ijcard.2005.12.027

[12] Lepoivre T, Legendre E, Pinaud M (2002) Anesthesia for cesarean section in a patient with McArdle disease and hereditary dilated cardiomyopathy. Ann Fr Anesth Reanim 21(6):517–520. 10.1016/S0750-7658(02)00645-7 12134596 10.1016/s0750-7658(02)00645-7

[13] Wang LW, Granger EK, McCourt JA, Pye R, Kaplan JM, Muller DWM (2015) Late surgical explantation and aortic valve replacement after transcatheter aortic valve implantation. Ann Thorac Surg 99(4):1434–1436. 10.1016/J.ATHORACSUR.2014.06.099 25841830 10.1016/j.athoracsur.2014.06.099

[14] Gandhi SE et al (2021) The phenotypic and genotypic features of a Scottish cohort with McArdle disease. Neuromuscul Disord 31(8):695–700. 10.1016/J.NMD.2021.05.009 34215481 10.1016/j.nmd.2021.05.009

[15] Lobato EB, Janelle GM, Urdaneta F, Malias MA (1999) W CASE REPORTS Noncardiogenic Pulmonary Edema and Rhabdomyolysis after Protamine Administration in a Patient with Unrecognized McArd le’s Disease. Available: http://pubs.asahq.org/anesthesiology/article-pdf/91/1/303/397584/0000542-199907000-00039.pdf 10.1097/00000542-199907000-00039 10422956

[16] Bollig G, Mohr S, Ræder J (2005) McArdle’s disease and anaesthesia: Case reports. Review of potential problems and association with malignant hyperthermia. Acta Anaesthesiologica Scandinavica, Blackwell Munksgaard 49(8):1077–1083. 10.1111/j.1399-6576.2005.00755.x. Blackwell Munksgaard 10.1111/j.1399-6576.2005.00755.x 16095447

[17] Scalco RS et al (2020) Data from the European registry for patients with McArdle disease and other muscle glycogenoses (EUROMAC). Orphanet J Rare Dis 15:1. 10.1186/s13023-020-01562-x 33234167 10.1186/s13023-020-01562-x PMC7687836

[18] Safiri S et al (2022) Burden of ischemic heart disease and its attributable risk factors in 204 countries and territories, 1990–2019. Eur J Prev Cardiol 29(2):420–431. 10.1093/eurjpc/zwab213 34922374 10.1093/eurjpc/zwab213

[19] Haller RG, Clausen T, Vissing J (1998) Reduced levels of skeletal muscle Na+K+-ATPase in McArdle disease 10.1212/wnl.50.1.37 9443454

[20] Ratinov G, Baker WP, Swaiman KF. CASE STUDIES McArdle’s Syndrome with Previously Unreported Electrocardiographic and Serum Enzyme Abnormalities. Available: https://annals.org 10.7326/0003-4819-62-2-328 14259215

[21] Molares-vila A, Corbalán-rivas A, Carnero-gregorio M, González-cespón JL, Rodríguez-cerdeira C (2021) Biomarkers in glycogen storage diseases: an update. Int J Mol Sci 22:9. 10.3390/ijms22094381 10.3390/ijms22094381 PMC8122709 33922238

