# Supplementary material for: Cardiac comorbidities in McArdle disease: case report and systematic review
Source: Neurol Sci. 2024 May 27;45(10):4757–65. doi: 10.1007/s10072-024-07600-x (PMC11422453; doi:10.1007/s10072-024-07600-x)
Supplement: Supplementary file 1 — Additional file 1: Table_S2. Quality Appraisal of Case Reports. Table_S3. Quality Appraisal of Original Articles. [file 10072_2024_7600_MOESM1_ESM.docx]

**Supplementary materials**

Table_S2: Quality Appraisal of Case Reports:

| Sr No | Clear Description of Demographic characteristics | Clear Description of history and presentation as a timeline | Clear Description of current clinical condition of patient | Clear Description of diagnostic tests or assessment methods | Clear description of intervention or treatment procedure | Clear description of post-intervention clinical condition | Identification of Adverse events or unanticipated events | Take away lessons | Score | Inclusion/Exclusion | Additional Comments |
| --- | --- | --- | --- | --- | --- | --- | --- | --- | --- | --- | --- |
| Nicholls et al. 1996 ^5^ | 1 | 1 | 1 | 1 | 1 | 1 | 1 | 1 | 8 | Included | None |
| Moustafa et al. 2012 ^6^ | 1 | 1 | 1 | 1 | 1 | 0 | 0 | 1 | 6 | Included | No drugs effects and adverse event |
| Marco-Benedí et al. 2019 ^7^ | 1 | 1 | 1 | 0 | 1 | 1 | 1 | 1 | 7 | Included | No diagnostic methods for miopathy |
| Jones et al. 2019 ^8^ | 1 | 1 | 0 | 1 | 1 | 0 | 1 | 1 | 7 | Included | No clinical neurological features |
| Vavouranakis et al. 2007 ^9^ | 1 | 1 | 1 | 1 | 1 | 1 | 1 | 1 | 8 | Included | None |
| Lepoivre et al. 2022 ^10^ | 1 | 1 | 1 | 0 | 1 | 1 | 1 | 1 | 7 | Included | No genetic of CMD and myopathy |
| Wang et al. 2015 ^11^ | 1 | 0 | 1 | 0 | 1 | 1 | 1 | 1 | 7 | Included | Absent description of muscular features and neurologic diagnostic methods |

Table_S3: Quality Appraisal of Original Articles:

| Sr No | Clear Description of Demographic characteristics | Clear Description of history and presentation as a timeline | Clear Description of current clinical condition of patient | Clear Description of diagnostic tests or assessment methods | Clear description of intervention or treatment procedure | Clear description of post-intervention clinical condition | Identification of Adverse events or unanticipated events | Take away lessons | Score | Inclusion/Exclusion | Additional Comments |
| --- | --- | --- | --- | --- | --- | --- | --- | --- | --- | --- | --- |
| Gandhi et al. 2021 ^13^ | 1 | 1 | 1 | 1 | 1 | 1 | 1 | 1 | 8 | Included | None |
